# Optimizing matching and analysis combinations for estimating causal effects

**DOI:** 10.1038/srep23222

**Published:** 2016-03-16

**Authors:** K. Ellicott Colson, Kara E. Rudolph, Scott C. Zimmerman, Dana E. Goin, Elizabeth A. Stuart, Mark van der Laan, Jennifer Ahern

**Affiliations:** 1 Division of Epidemiology, University of California- Berkeley School of Public Health, 50 University Hall, Berkeley, CA 94720-7360, USA; 2 Center for Health and Community, University of California- San Francisco, 3333 California St, Suite 465, San Francisco, CA 94143-0844, USA; 3 Departments of Mental Health, Biostatistics, and Health Policy and Management, Johns Hopkins Bloomberg School of Public Health, 624 N Broadway, Baltimore, MD 21205, USA; 4 Division of Biostatistics, University of California- Berkeley School of Public Health, 101 Haviland Hall, Berkeley, CA 94720-7358, USA.

## Abstract

Matching methods are common in studies across many disciplines. However, there is limited evidence on how to optimally combine matching with subsequent analysis approaches to minimize bias and maximize efficiency for the quantity of interest. We conducted simulations to compare the performance of a wide variety of matching methods and analysis approaches in terms of bias, variance, and mean squared error (MSE). We then compared these approaches in an applied example of an employment training program. The results indicate that combining full matching with double robust analysis performed best in both the simulations and the applied example, particularly when combined with machine learning estimation methods. To reduce bias, current guidelines advise researchers to select the technique with the best post-matching covariate balance, but this work finds that such an approach does not always minimize mean squared error (MSE). These findings have important implications for future research utilizing matching. To minimize MSE, investigators should consider additional diagnostics, and use of simulations tailored to the study of interest to identify the optimal matching and analysis combination.

Matching is a common approach to address confounding, particularly in studies that utilize observational data. Matching methods estimate the effect of a treatment, program, or policy by comparing treated (or exposed) units to control (or unexposed) units with similar observed characteristics. This approach is based on the potential outcomes^1^ and other causal inference frameworks^2^,^3^,^4^. Use of matching has increased as the corresponding methodological groundwork has developed^5^,^6^,^7^. There are many types of matching methods^8^,^9^,^10^,^11^ that have been used across scientific disciplines, including statistics^4^, economics^7^,^12^, political science and public policy analysis^13^,^14^, criminology^15^,^16^, education^17^,^18^, sociology^19^,^20^, psychology and behavioral health^21^,^22^, and medicine and public health^23^.

Matching methods help address differences in the distributions of measured covariates between treated and control groups by improving the balance of these factors, as would be expected in a randomized experiment. They also facilitate comparisons between study groups with good support by encouraging the researcher to only compare covariate subgroups that have both treated and control units, thereby avoiding extrapolation^8^,^9^,^24^. While covariate support, also known as positivity, should be examined in any analysis, this is not an inherent step in many regression-based methods^11^,^25^. Matching is also intuitive^6^,^8^ and may make fewer assumptions about the forms of the relationships between covariates, treatment, and outcome (e.g., allows for non-linearity) than standard regression analyses^8^,^9^. After matching, additional analyses can be applied to improve the estimate of effect.

Despite the large and multidisciplinary literature on matching methods, there is no consensus on how matching should be executed or evaluated. There are many general guidelines—for example, minimum covariate balance thresholds^9^,^26^ or weight trimming when support is poor^10^—some of which contradict one another or are ambiguous. To select among the myriad matching procedures, current guidelines commonly advise researchers to examine the balance of covariates between the treated and control groups and to select the matching approach that achieves the best post-matching balance (see for example^11^,^27^). One basis for this recommendation is the logic of a randomized experiment in which covariate balance is used to assess whether randomization has been effective and hence, whether bias is likely to be minimized^28^. While some suggest that balance may be a good indicator of bias, and existing work has shown its value for that purpose^11^,^29^,^30^,^31^, it is not known how well this recommendation optimizes mean squared error (MSE), a measure of performance that incorporates both bias and variance. Several previous studies have compared the performance of different matching methods (see for example^32^,^33^,^34^). However, these studies are limited in scope; all are either restricted to propensity score-based methods, study the performance of matching estimators without subsequent analysis, include only a small set of matching methods or analyses, or evaluate performance based on bias alone, not bias, variance, and MSE. It has not been systematically examined how balance measures relate to overall performance (MSE) of effect estimates, when considering a wide range of matching and analysis techniques. Furthermore, no previous studies have included systematic comparisons of matching coupled with double robust or semi-parametric estimation methods, which have shown superior performance in many settings^35^,^36^,^37^.

We conducted simulation studies to compare the performance of a wide range of matching and analysis combinations in estimating the average effect of treatment on the treated (ATT). The ATT is relevant when one is interested in the effect of an exposure among those who are exposed. For example, when studying an employment training program, the effect of the program on the unemployed people who received it would be more relevant than the effects in the general population, most of whom would already be employed. Similarly, with a harmful exposure such as drug use, the effect of drug use on health in those who use drugs is more interpretable than an effect in a general population many of whom have never used drugs.

Simulation-based approaches to compare estimation performance are appealing because the true effect of interest is known and can therefore be compared to estimates generated from different statistical methods under varying conditions. We considered whether covariate balance, assessed using recommended metrics^11^,^31^,^38^,^39^, identified the matching and analysis combination with the lowest MSE in the treatment effect estimate. Then, using data from a randomized employment training program, we compared the experimental effect estimate to effect estimates generated by applying matching and analysis combinations to data with observational controls drawn from a general population survey.

## Methods

### Simulated data

We simulated 1,000 data sets of size 1,000, comprised of a continuous outcome Y, a binary indicator of treatment A, and two baseline covariates W1 and W2. In this paper, we use the terms “treated” and “control” to refer to groups we wish to compare, but relevant studies need not involve an explicit treatment as in biomedical research. The simulations were designed to imitate data that could realistically arise in observational settings and to demonstrate the performance of combinations of matching and analysis methods in the presence of treatment effect heterogeneity and confounding of the relationship between the treatment and the outcome. Specifically, W1 and W2 were uniform and normal random variables, respectively. Treatment A was binomial with probability of success dependent on W1 and W1, and outcome Y was random normal with mean dependent on W1, W1, and A. Dependencies included squared terms and multiplicative interactions. The complete data generating mechanisms are presented in the Supporting Information (SI) Text.

### Employment program data

As an applied example, we use data originating from LaLonde’s 1986^40^ study of the National Supported Work (NSW) Demonstration, a large-scale employment training program that aimed to increase income levels by providing work experience and counseling to individuals who lacked basic job skills. Applicants were randomly assigned to the NSW program, or to the control group. Data on participants and controls was collected at baseline and at up to four post-baseline time points using surveys and Social Security Administration records. The outcome of interest was real earnings in 1978 and baseline covariates were age, years of education, high school completion, black race, Hispanic ethnicity, marital status, and real earnings in 1974 and 1975.

We used the publicly available dataset constructed by Dehejia and Wahba^7^, which includes both the experimental data and observational population-based controls. This arrangement allows researchers to compare effect estimates from the randomized data to estimates that might have been generated by comparing outcomes for individuals participating in the program to general population controls (an observational study design), had the randomized trial not been executed. The experimental data include 185 participants and 260 controls. The observational controls were drawn from Westat’s Matched Current Population Survey-Social Security Administration file containing 15,992 general population controls. Additional information on the NSW program and the dataset used in this study are available elsewhere^7^,^40^.

### Estimation methods

We estimated the average effect of treatment on the treated (ATT) in both the simulated data and applied example, by applying seven matching approaches, three analysis methods, and two estimation approaches (parametric and semi-parametric). The ATT estimand is the average difference between potential outcomes^2^ for the exposed units under exposure, and the exposed units had they been unexposed. The methods estimate the ATT by comparing the average outcome in the exposed group to the average outcome in a comparison group of unexposed units that has been selected, weighted, or otherwise adjusted to approximate the covariate distribution of the exposed units. The matching and analysis methods, described in greater detail below, relied on estimation of the treatment mechanism, or propensity score^25^, and the outcome model. We estimated these models in two ways: First, parametrically, by assuming a functional form (main terms only) and applying linear or logistic regression, and second, semi-parametrically, by applying the SuperLearner ensemble machine learning algorithm^35^. While parametric approaches are standard and far more common in practice, recent evidence suggests that semi-parametric approaches may reduce bias and increase efficiency^35^,^41^,^42^.

When analyzing the simulated data, we assumed parametric model forms that were misspecified given the data generating mechanism. This is because correct specification of the model form is unlikely in applied settings where the true underlying data generating mechanisms are unknown. Hence, the analysis aligns with what is done in practice. It further provides an opportunity to examine potential gains from semi-parametric estimation when the model form is not known.

### Matching methods

Using the framing of Ho *et al.*^9^, we considered each matching procedure as a form of pre-processing, after which the ATT could be estimated by calculating the difference of mean outcomes between treated and controls units (a “naïve” analysis) or applying further analysis techniques. The matching approaches were: one-to-one greedy nearest neighbor (NN) matching with replacement^43^; one-to-one optimal nearest neighbor (optimal) matching without replacement^44^; subclassification (with ten propensity score quantiles in simulations and five quantiles in the applied example)^24^; full matching^4^,^45^; inverse probability of treatment weighting (IPTW)^25^,^46^; and genetic matching^38^. A detailed description of each method can be found in the Supporting Information. We also considered unmatched data.

The matching methods relied on measures of the distance between covariate values in the treatment and control groups. In all cases except genetic matching, this distance metric was the propensity score, estimated parametrically and semi-parametrically, as described above. In the case of genetic matching, the distance measure was the generalized Mahalanobis metric, as recommended^38^. Results for the parametric and semi-parametric matching approaches were very similar. For parsimony, we present the parametric version in the main text and the results for all tested simulations in SI Tables S1–S3.

### Analysis methods

Estimators of the ATT are available that adjust for covariates based on the treatment mechanism, the outcome mechanism, or both (also known as double-robust methods). After a matching approach, which utilizes the treatment mechanism, is applied, an analysis approach is used to compare outcomes in the matched samples. We considered three outcome analyses: a naïve analysis, g-computation^46^, and targeted minimum loss-based estimation (TMLE)^47^. G-computation is a maximum likelihood based substitution estimator of the G-formula. It is implemented by using regression to model the outcome as a function of the exposure and covariates. The fitted model is then used to predict the outcome under different exposure scenarios to be compared. To estimate the ATT, we average the difference between the model predictions for all exposed units had they been unexposed and the model predictions for all exposed units had they been exposed. Typically, g-computation relies on a parametric model. TMLE for the ATT is general two-stage efficient substitution estimator. In the first stage, we model the outcome as a function of the exposure and covariates. The second stage is a bias reduction step that iteratively updates the parameter estimates using models of the exposure given covariates (the treatment mechanism). This updating step also makes the estimator double-robust, asymptotically normal, and asymptotically efficient. TMLE is typically implemented with semi-parametric machine learning methods. Treatment and outcome models for TMLE were estimated using parametric and semi-parametric approaches, as described above. For g-computation, we used only parametric estimation, as inference using semi-parametric g-computation has not been established.

### Balance and performance metrics

For each matched sample we calculated a large set of balance metrics recommended in the literature^11^,^31^,^38^,^39^. These included: the mean, median, and maximum absolute standardized mean difference (ASMD) in covariate values between the treated and control groups across all eight covariates, the percent of covariates with ASMD less than 20%, 10%, 5%, and 1%, the ASMD of the prognostic score (a formulation of the disease risk score^39^), the ASMD of the propensity score, and the absolute mean t-statistic comparing covariate values between the treatment and control groups.

The propensity scores and prognostic scores used to measure balance were distinct from those used to estimate effects. In the simulated data, the propensity scores and prognostic scores used to measure balance were quantified using generalized linear regression and the known treatment and outcome model forms (see SI Text). In the NSW data, the propensity scores and prognostic scores used to measure balance were estimated using main terms logistic regression and SuperLearner. In the NSW applied example, the propensity scores and prognostic scores were indistinguishable across the two estimation procedures, so only the logistic regression results are reported. We also present plots of the distributions of propensity scores by treated and control groups to illustrate the degree of covariate support in the different scenarios^25^,^48^.

For each data generating mechanism, we created 1,000 simulated datasets and evaluated the performance of each matching and analysis combination. The primary measure of performance was the mean squared error (MSE = bias^2^ + variance), where lower MSE indicates a better estimate. We also compared the average percent bias and the variance. We present all three performance measures for each of the estimators considered. To estimate the bias, variance, and MSE of the effect estimates in the applied example and to account for stochastic elements of the matching and machine learning algorithms, we analyzed 500 bootstrapped datasets^49^,^50^,^51^.

Additional information on the simulations, applied example, matching methods, and analyses are available in SI Text. All analyses were conducted in R version 3.2.0^52^. Matching was implemented using the MatchIt package^53^, and its dependent packages. TMLE was implemented using a modified version of the tmlecte package (to incorporate matching weights) which is available on Github^54^.

## Results

### Simulation results

**Figure 1:**
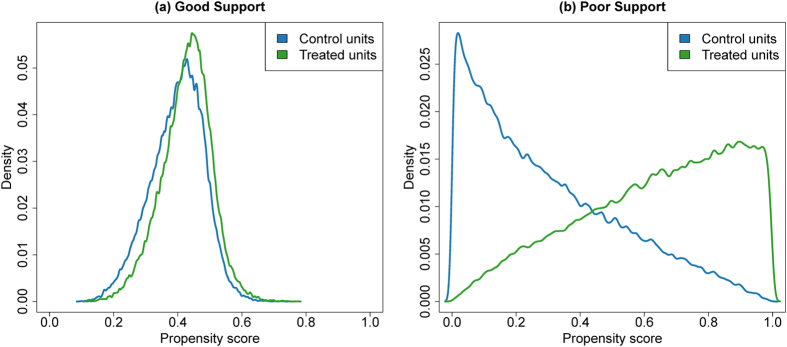
(**a**) Density of estimated propensity scores for treated and control units in good support scenario. (**b**) Density of estimated propensity scores for treated and control units in poor support scenario. The plots illustrate substantial overlap of the propensity scores for treated and control units in the good support scenario and minimal overlap in the poor support scenario. The probability of treatment ranged between 0.093 and 0.776 in the good support scenario and between <0.001 and >0.999 in the poor support scenario. Distributions and results for the medium support scenario fell in between those of the good and poor support scenarios and are presented in SI Text, SI Fig. S1, and SI Table S2.

The distributions of the propensity score, by treated and control units, are presented in Fig. 1. The plots correspond to the scenarios of good support (substantial overlap of the propensity scores for treated and control) and poor support (minimal overlap). The probability of treatment ranged between 0.093 and 0.776 in the good support scenario and between <0.001 and >0.999 in the poor support scenario. Distributions and results for the medium support scenario fell in between those of the good and poor support scenarios and are presented in SI Text, SI Fig. S1, and SI Table S2.

Selected balance metrics for the good and poor support scenarios are presented in Table 1 (see SI Table S4 for all support scenarios and balance metrics). Across all of the simulation scenarios, genetic matching consistently resulted in the best covariate balance, according to all balance metrics. However, when combined with analysis methods, genetic matching did not generally result in the ATT estimate with the lowest MSE.

In the good support scenario, many matching and analysis methods performed well and there were few substantive differences between estimators in terms of the MSE (Table 2). Across the 28 methods, 68% had MSE less than 0.01 and 75% were less than 1% biased. In terms of MSE, full matching with parametric TMLE performed best, and five of the top ten methods involved double robust analysis (TMLE with parametric or semi-parametric estimation). Subsequent analysis after matching generally improved performance, compared with matching alone. Several of the top-performers also involved no matching at all. In terms of MSE, full matching, IPTW, subclassification, and optimal matching were higher performing matching methods, while genetic and greedy NN matching were lower performing matching methods. Semi-parametric estimation for TMLE did not substantively improve performance over parametric TMLE. When considering only bias, full, NN, and genetic matching were high performers. The lowest bias was achieved for NN matching paired with semi-parametric double robust analysis.

In the poor support scenario (Table 3), fewer methods performed well. Across the 28 methods, 7% had MSE less than 0.01 and 29% were less than 1% biased. Again, full matching with TMLE was the top-performer in terms of MSE, and the top five methods involved double robust analysis. However, in this scenario, semi-parametric estimation for TMLE notably improved performance over parametric TMLE, except when paired with subclassification. There was again a clear benefit to utilizing matching and subsequent analysis together, as methods with no matching or naïve analysis generally performed poorly. An exception to this pattern was no matching with semi-parametric TMLE, which performed relatively well and had the lowest bias. In contrast with the good support scenario, genetic matching was a mid- to high-performer. The top four methods with the lowest bias involved semi-parametric TMLE, making this approach one of the most consistent performers for bias reduction across all scenarios.

### Employment intervention results

**Figure 2:**
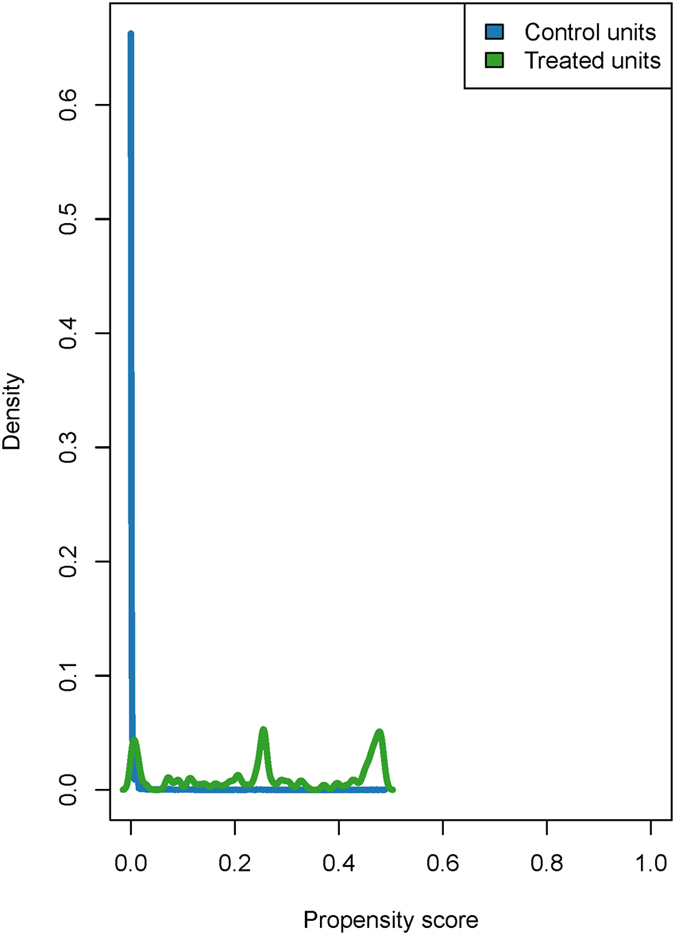
Density of estimated propensity scores for treated experimental units and observational control units. The plot illustrates extremely poor overlap of the propensity scores for treated and control units in the applied example. Propensity scores ranged between <0.001 and 0.488, and were substantially skewed towards 0 for the control group. These patterns indicate that the baseline characteristics of the control individuals are very different from those who participated in the program; this example is most similar to the poor support simulation scenario.

As an applied example, we compared the experimental results from the National Supported Work (NSW) employment program to those estimated using observational controls. Based on the experimental data, the NSW program increased real earnings of those in the treatment group by an average of $1,794 (comparison of means). We treat this quantity as the true effect of the program. In analyses combining the experimental treatment group with observational control data, the level of covariate support before matching was extremely poor. The distribution of propensity scores by treated and control units are presented in Fig. 2. Propensity scores ranged between <0.001 and 0.488, and were substantially skewed towards 0 for the control group. These patterns indicate that the baseline characteristics of the control individuals are very different from those who participated in the program; this example is most similar to the poor support simulation scenario.

As in the simulations, genetic matching generally resulted in the best covariate balance in the NSW observational data, but not for all metrics (see Table 4 for summary; see SI Table S5 for all metrics). Six of ten metrics indicated that genetic matching generated the best balance, while three others indicated that IPTW generated the best balance, and one indicated that full matching achieved the best balance (SI Table S5). In cases where genetic matching did not achieve the best balance, the metrics were generally very close to those of the best method. As in simulations, genetic matching did not result in the matching and analysis combination with the lowest MSE.

**Figure 3:**
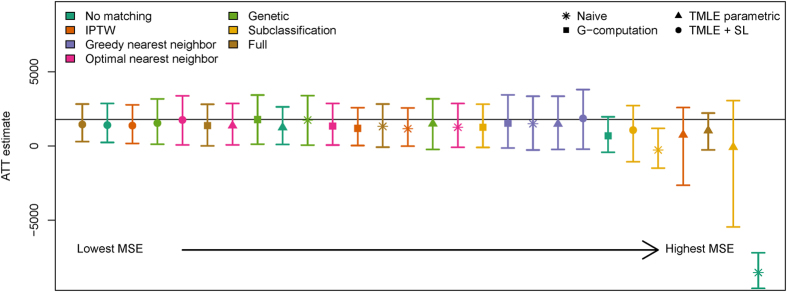
Comparison of matching and analysis combinations to estimate the effect of NSW participation using observational control data. Colored points represent point estimates of the effect of treatment on the treated (ATT), with corresponding 95% error bars. The unadjusted estimate of the ATT in the NSW observational data was -$8,526, dramatically different from the experimental result of $1,794 (indicated by the grey line). The success of matching and analysis combinations in recovering the experimental result varied substantially. Almost all methods underestimated the experimental result, suggesting a consistent residual bias that may be the result of unmeasured covariates. The confidence intervals for all ATT estimates were wide, and few estimates excluded the null. IPTW: inverse probability of treatment weighting. TMLE: targeted minimum loss-based estimation. SL: using SuperLearner for semi-parametric estimation.

The unadjusted estimate of the ATT in the NSW observational data was -$8,526, dramatically different from the experimental result of $1,794. The success of matching and analysis combinations in recovering the experimental result varied substantially (see Fig. 3 and SI Table S6). As in the simulations, the methods with the lowest MSE were full matching, genetic matching, optimal matching or IPTW paired with semi-parametric TMLE. While semi-parametric TMLE was involved in the top five performing methods, full, genetic, and optimal matching paired with other analyses also performed well. Subclassification and greedy nearest neighbor matching fared poorly. Interestingly, analyses involving no matching were among both the best and the worst, depending on the analysis with which they were paired. Genetic matching with g-computation was the least biased method. Almost all methods underestimated the experimental result, suggesting a consistent residual bias that may be the result of unmeasured covariates. The confidence intervals for all ATT estimates were wide, and few estimates excluded the null.

## Discussion

We evaluated the performance of a wide variety of matching and analysis methods in estimating the ATT in a simulation study and applied example. The best-performing method depended on the degree of covariate support. When support was good, many matching and analysis approaches generated estimates with minimal bias and low variance. The high performance of double robust methods without matching (TMLE) indicated that matching may not provide a meaningful advantage when support is good. In contrast, when support was poor, fewer methods performed well, and the benefits of combining matching with double robust analysis, especially when incorporating machine learning for estimation, became more prominent. The combination of full matching and semi-parametric TMLE was the most consistently high-performing method in both the simulations and the applied example. Therefore, we expect this combination would perform well in other scenarios as well. The advantages of these methods in the poor support scenario are of particular interest, because settings of poor support are common in applied, observational research^55^,^56^,^57^.

While we studied many novel matching and analysis combinations that had not been studied previously, some of our findings are consistent with previous research. Schafer and Kang^58^,^59^ and Radice and colleagues^34^ used simulations to compare several different matching and analysis methods, and found that in good support scenarios, many analyses reported similar bias, variance, and MSE, but under poor support, there was significant heterogeneity in performance. Radice and colleagues also found that genetic matching achieved better balance than several other matching methods. At least two other studies^29^,^30^ found that full matching resulted in lower bias, but to our knowledge, none have examined the relationship with MSE. Estimators that combine matching with further adjustment^60^,^61^,^62^,^63^ have also been shown to offer performance benefits in previous research.

Previous research has demonstrated advantages of double robust analysis and semi-parametric estimators^32^,^36^,^47^,^64^, but few studies have evaluated how machine learning may improve estimates of effect when combined with any matching method^38^,^41^,^42^, let alone a broad range of matching methods. We are the first to systematically combine double robust methods with matching techniques and to consider semi-parametric estimation at every stage. Our results make an important contribution to the literature, because they indicate that these novel combinations may offer additional performance advantages over any individual analytic technique previously assessed. We found that use of the matching method with the best covariate balance does not necessarily lead to the estimator with the best overall performance, as measured by the MSE. Across simulations and an applied example, genetic matching achieved the best balance, but analyses that included genetic matching had larger MSE than the top-performing method in all cases. Two previous studies found that genetic matching achieved both better balance and lower MSE than other propensity score-based methods^34^,^38^, but they compared a limited number of estimators, and did not consider matching in combination with any subsequent analysis. We add to a small number of studies that caution against using of covariate balance as the only criteria for selecting a matching approach (see for example^28^,^41^,^65^).

Much of the previous literature on matching emphasized covariate balance for minimizing bias rather than MSE (see for example^43^,^62^,^66^,^67^,^68^). Indeed, in our simulations, we found that methods with better covariate balance were generally less biased. If a researcher’s goal is to minimize MSE, further investigation is needed to identify additional diagnostics that are more closely related to the combination of bias and variance. As might be expected, the balance measures we examined had little to do with variance, and hence, were imperfect indicators of MSE. While the balance metrics we considered are common in the matching literature^11^,^31^,^38^,^39^, most involve comparing single summary measures of univariate distributions. Ideal measures of balance would allow one to compare the full multivariate distributions of the covariates, and ideal diagnostics for MSE would incorporate both balance in multivariate distributions and efficiency of different estimators.

Some matching methods with good balance may not perform as well in terms of MSE due to differences in effective sample size. Because some matching methods drop observations, while others keep all observations and reweight, they may have very different effective sample sizes. Genetic matching is designed to optimize covariate balance, not MSE, and in process may drop observations and thereby sacrifice statistical precision^5^,^9^,^69^. In contrast, full matching retains all observations while maintaining covariate balance that is only slightly worse than that of genetic matching. This tradeoff between balance, bias, and statistical precision must be considered carefully given the specific goals of each unique study.

In this study, we identified full matching combined with double robust analysis and semi-parametric estimation as a high-performing estimator across numerous scenarios. However, it is possible that in other scenarios, the best-performing methods would be different. For example, the performance of parametric models will depend on the degree of model misspecification; in this work, we examined one type of misspecification, but future work should investigate how performance is altered under varying misspecification scenarios. The variable results of previous comparisons of matching methods attest that there is no single approach that best fits all scenarios (see for example^38^,^29^,^32^). Our study, along with others (for example^60^,^61^), illustrates the utility of simulation in determining the optimal analysis. In any applied study, the true effect is unknown, but researchers can use simulations to inform the choice of analytic method, supplementing general guidelines, which may be based on scenarios quite different from the one at hand. Ideally, the choice of matching and analysis for every study would be informed by simulation that reflects the setting and study question. The use of simulation to inform methodological choices is not new^43^,^62^,^66^. However, it is rarely used to inform choices in applied research. Fortunately, recognition of the value of simulation studies is increasing, and implementing simulations is becoming increasingly accessible with the release of new R packages^70^ and web-based software with graphical user interfaces^71^,^72^. The use of these tools can help researchers to make more rigorous and tailored decisions about study design and analysis approaches.

Our study has several limitations. We considered a broad range of commonly applied matching techniques, together with an array of analysis methods, with particular focus on incorporation of double robust analysis and semi-parametric estimation approaches. However, these are a subset of the possible matching and analysis combinations one could consider. Likewise, there are other balance metrics or estimands we could have considered. For example, we might have considered coarsened exact matching, augmented IPTW, the joint significance and pseudo R^2^ for measuring balance, estimators of the average treatment effect (ATE), or estimators for longitudinal data structures. However, the subset we have chosen clearly demonstrates the advantages of semi-parametric and double robust methods not previously examined, and further, illustrates that balance is not the best indicator of estimator performance in terms of MSE. There are no agreed upon best measures of balance^11^, and those we did examine displayed consistent results. A simulation approach similar to the one we have taken would be well-suited to identify optimal approaches for other matching methods, balance metrics, estimands, or data generating mechanisms.

To estimate variance, we utilized non-parametric bootstrapping. This approach is widely used to approximate the distribution of point estimates and to quantify uncertainty^49^. However, minimal previous scientific work has examined the validity of bootstrapping for inference for the unique combinations of matching analysis procedures included in this paper. Further methodological development is needed in the areas of variance estimation for estimators involving matching. Simulated data are unlikely to possess the unusual distributional properties sometimes present in real data. For this reason, some simulation studies employ covariates from real data and simulate only the outcome^61^. However, our simulation results are bolstered by the use of real data from the evaluation of the NSW employment training program; the similarity in results between the simulations and applied example increase our confidence that the simulation results are relevant to real world scenarios.

In this study, we identified optimal combinations of matching and analysis methods for estimates of the ATT. We presented a simulation study and applied example to quantify the performance of a range of combinations of matching and analysis approaches. Our findings demonstrated the superior performance of novel combinations of matching with double robust analysis and semi-parametric estimation. In particular, full matching combined with semi-parametric TMLE was a consistent top-performer, particularly when support was poor. In addition, we concluded that selecting an approach based on the currently recommended balance metrics may not lead to the least biased and most efficient estimate. We call for the development of more sophisticated balance metrics and other diagnostics that better align with performance in terms of MSE and for the increased use of tailored simulation to inform the choice of analytic methods. This would support the systematic selection of methods with better performance, rather than the methods that are most familiar or easy to implement. Such an approach has the potential to improve the rigor and quality of studies across a broad array of disciplines.

## Additional Information

**How to cite this article**: Colson, K. E. *et al.* Optimizing matching and analysis combinations for estimating causal effects. *Sci. Rep.* **6**, 23222; doi: 10.1038/srep23222 (2016).

## Supplementary Material

Supporting Information

Supporting Information Sample Code 1

Supporting Information Sample Code 2

## Figures and Tables

**Table 1:** Balance metrics by simulation scenario and matching method.

Match method	Percent of covariates with ASMD less than…		Median ASMD	Maximum ASMD	ASMD in propensity score
	20%	5%			
Good support					
None	46.1	5.3	0.214	0.443	0.362
NN	100	80.6	0.024	0.147	0.072
Opt	100	81.1	0.025	0.148	0.069
Genetic	**100**	**100**	**0.000**	**0.024**	**0.006**
Sub	100	99.7	0.013	0.055	0.048
Full	100	94.7	0.014	0.092	0.054
IPTW	100	99.7	0.013	0.072	0.068
Poor support					
None	48.1	13.8	0.726	0.941	1.184
NN	74.4	36.5	0.090	0.330	0.126
Opt	49.6	20.7	0.544	0.822	0.953
Genetic	**99.1**	**64.6**	**0.042**	**0.220**	**0.038**
Sub	78.9	7.4	0.131	0.451	0.132
Full	79.0	36.3	0.111	0.335	0.145
IPTW	62.5	15.1	0.130	1.643	0.200

ASMD: Absolute standardized mean difference in covariate values between treated and control groups. NN: greedy nearest neighbor. Opt: optimal nearest neighbor. Sub: subclassification. IPTW: inverse probability of treatment weighting. Metrics are averaged across 1,000 simulation runs. Bolded values indicate the best balance according to each metric and scenario.

**Table 2:** Simulation results comparing matching and analysis combinations in good support scenario.

Match	Analysis	% Bias	Var	MSE	Bias rank	Var rank	MSE rank
Full	TMLE parametric	0.28%	0.0063	0.0063	16	2	1
None	TMLE with SL	−0.02%	0.0065	0.0065	3	3	2
None	TMLE parametric	−0.14%	0.0066	0.0066	11	4	3
IPTW	g-computation	0.13%	0.0066	0.0066	9	5	4
IPTW	TMLE with SL	0.02%	0.0067	0.0067	2	6	5
Sub	g-computation	−0.48%	0.0067	0.0067	19	7	6
IPTW	Naïve	−0.52%	0.0068	0.0069	20	9	7
Sub	TMLE with SL	−0.45%	0.0069	0.0070	18	10	8
Opt	g-computation	−1.35%	0.0067	0.0071	23	8	9
Sub	Naïve	0.89%	0.0070	0.0072	21	11	10
Opt	TMLE with SL	−0.08%	0.0072	0.0072	6	13	11
Full	TMLE with SL	−0.04%	0.0073	0.0073	4	14	12
Opt	TMLE parametric	−1.25%	0.0071	0.0074	22	12	13
Full	g-computation	−0.21%	0.0077	0.0077	12	16	14
Full	Naive	0.24%	0.0080	0.0081	14	17	15
Sub	TMLE parametric	−1.52%	0.0082	0.0087	24	18	16
Genetic	TMLE with SL	0.11%	0.0090	0.0090	7	19	17
Genetic	g-computation	0.05%	0.0093	0.0093	5	20	18
Genetic	Naive	0.14%	0.0093	0.0093	10	21	19
NN	TMLE with SL	0.02%	0.0102	0.0102	1	23	20
Opt	Naive	2.23%	0.0097	0.0107	25	22	21
NN	g-computation	−0.23%	0.0107	0.0107	13	24	22
NN	Naive	0.28%	0.0113	0.0113	15	25	23
Genetic	TMLE parametric	0.33%	0.0115	0.0115	17	26	24
NN	TMLE parametric	−0.12%	0.0129	0.0129	8	27	25
None	g-computation	−8.51%	0.0054	0.0202	27	1	26
IPTW	TMLE parametric	7.97%	0.0077	0.0207	26	15	27
None	Naive	21.22%	0.0147	0.1073	28	28	28

Var: variance. MSE: mean squared error. NN: greedy nearest neighbor. Opt: optimal nearest neighbor. Sub: subclassification. IPTW: inverse probability of treatment weighting. SL: using SuperLearner for semi-parametric estimation.

**Table 3:** Simulation results comparing matching and analysis combinations in poor support scenario.

Match	Analysis	% Bias	Var	MSE	Bias rank	Var rank	MSE rank
Full	TMLE with SL	−0.81%	0.0088	0.0091	8	3	1
IPTW	TMLE with SL	−0.76%	0.0090	0.0092	6	4	2
Genetic	TMLE with SL	−0.65%	0.0130	0.0132	3	8	3
NN	TMLE with SL	−0.70%	0.0140	0.0141	4	9	4
Opt	TMLE with SL	0.53%	0.0166	0.0167	2	14	5
IPTW	G-computation	−0.70%	0.0169	0.0171	5	15	6
None	TMLE with SL	0.52%	0.0179	0.0179	1	16	7
Sub	Naive	3.47%	0.0158	0.0201	18	13	8
Genetic	g-computation	−0.78%	0.0210	0.0212	7	19	9
Full	g-computation	−1.55%	0.0204	0.0212	10	17	10
Full	Naive	1.77%	0.0210	0.0221	13	18	11
Genetic	Naive	1.59%	0.0216	0.0224	11	20	12
NN	g-computation	−1.82%	0.0217	0.0229	14	21	13
NN	Naive	1.76%	0.0222	0.0232	12	22	14
Opt	TMLE parametric	1.11%	0.0247	0.0251	9	24	15
Sub	g-computation	−3.46%	0.0243	0.0285	17	23	16
None	TMLE parametric	1.89%	0.0285	0.0297	15	25	17
Genetic	TMLE parametric	−6.69%	0.0145	0.0303	19	10	18
IPTW	TMLE parametric	−7.56%	0.0106	0.0308	22	5	19
NN	TMLE parametric	−7.22%	0.0147	0.0331	20	12	20
Full	TMLE parametric	−7.84%	0.0118	0.0335	23	7	21
IPTW	Naive	−1.94%	0.0493	0.0506	16	27	22
Sub	TMLE parametric	−7.50%	0.0467	0.0665	21	26	23
Sub	TMLE with SL	−8.04%	0.0512	0.0740	24	28	24
None	g-computation	−26.75%	0.0074	0.2605	25	1	25
Opt	g-computation	−28.68%	0.0074	0.2982	26	2	26
Opt	Naive	45.27%	0.0147	0.7393	27	11	27
None	Naive	60.41%	0.0110	1.3013	28	6	28

Var: variance. MSE: mean squared error. NN: greedy nearest neighbor. Opt: optimal nearest neighbor. Sub: subclassification. IPTW: inverse probability of treatment weighting. SL: using SuperLearner for semi-parametric estimation.

**Table 4:** Balance metrics for NSW observational data by matching method.

Match method	Percent of covariates with ASMD less than…		Median ASMD	Maximum ASMD	ASMD in propensity score
	20%	5%			
None	12.2	5.0	1.182	1.471	4.901
NN	94.4	45.1	0.066	0.309	0.490
Opt	98.6	56.2	0.048	0.227	0.255
Genetic	**100**	**93.5**	**0.008**	0.160	0.060
Sub	60.0	4.8	0.181	0.352	0.062
Full	97.9	58.3	0.048	0.215	**0.012**
IPTW	100	91.8	0.015	**0.048**	0.028

ASMD: Absolute standardized mean difference in covariate values between treated and control groups. NN: greedy nearest neighbor. Opt: optimal nearest neighbor. Sub: subclassification. IPTW: inverse probability of treatment weighting. Metrics are averaged across 500 bootstrapped samples. Bolded values indicate the best balance according to each metric and scenario.
