# Supplementary material for: Optimizing matching and analysis combinations for estimating causal effects
Source: Sci Rep. 2016 Mar 16;6:23222. doi: 10.1038/srep23222 (PMC4793248; doi:10.1038/srep23222)
Supplement: Supporting Information [file srep23222-s1.doc]

**Optimizing matching and analysis combinations for estimating causal effects**

K. Ellicott Colson ^a^, MPH

Kara E. Rudolph ^a,b^, PhD MPH MHS

Scott C. Zimmerman ^a^, MPH

Dana E. Goin ^a^, BS

Elizabeth A. Stuart ^c^, PhD

Mark van der Laan ^d^, PhD

Jennifer Ahern ^a^*, PhD MPH

**Supporting Information**

**Text**

**Materials and Methods**

***Simulated data***

We conducted a simulation study to compare performance of a large set of matching and analysis combinations in estimating the average treatment effect among the treated (ATT) ^1^ under several scenarios. Simulation-based approaches to compare estimation performance are appealing because the true effect of interest is known and can therefore be compared to estimates generated from different statistical methods under varying conditions and using a variety of analytic methods.

We simulated 1,000 data sets of size 1,000, comprised of a continuous outcome Y, a binary indicator of treatment A, and two baseline covariates W1 and W2. In this paper, we use the terms “treated” and “control” to refer to groups we wish to compare, but relevant studies need not involve an explicit treatment as in biomedical research. The simulations were designed to imitate data that could realistically arise in observational settings and to demonstrate the performance of combinations of matching and analysis methods in the presence of treatment effect heterogeneity and confounding of the relationship between the treatment and the outcome.

Specifically, covariates
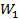
 and
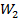
 were generated as uniform and normal random variables[[1]](#footnote-2) of the form


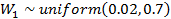


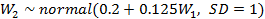


We tested three different exposure mechanisms of modest complexity (non-linear associations and interactions) with varying levels of covariate support (also known as practical positivity violations). Ordered from best support to poorest support, the treatment mechanisms were:


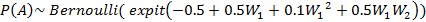
 (1)


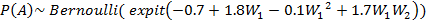
 (2)


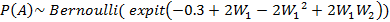
 (3)

We refer to the treatment mechanisms as the good, medium, and poor support scenarios, respectively. The outcome
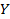
 was normally distributed of the form


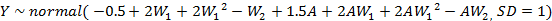


Note that both
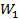
 and
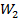
 are confounders and that all three scenarios introduce substantial bias in the unadjusted ATT (21%, 40%, and 60%, respectively).

***Employment program data***

As an applied example, we use data originating from LaLonde’s 1986 ^2^ study of the effect of the National Supported Work (NSW) Demonstration, a large-scale employment training program. Implemented in the mid-1970s in 10 sites across the United States, the intervention aimed to increase income levels by providing work experience and counseling to workers who lacked basic job skills. Individuals facing significant social and economic hardship (those lacking a high school degree, with prior involvement in criminal justice system, recovering from drug abuse) were eligible for enrollment. Applicants were randomly assigned to the NSW program, or to the control group, which offered no further intervention. Data on participants and controls was collected at baseline and at up to four post-baseline time points using surveys and Social Security Administration records. The outcome of interest was real earnings in 1978 and baseline covariates were age, years of education, high school completion, black race, Hispanic ethnicity, marital status, and real earnings in 1974 and 1975.

We used the publicly available dataset constructed by Dehejia and Wahba ^3^, which includes both the experimental data and observational population-based controls. This arrangement allows researchers to compare effect estimates from the randomized data to estimates that might have been generated by comparing outcomes for individuals participating in the program to general population controls (an observational study design), had the randomized trial not been executed. The experimental data include 185 participants and 260 controls. The observational controls were drawn from Westat’s Matched Current Population Survey-Social Security Administration file containing 15,992 general population controls. Additional information on the NSW program and the dataset used in this study are available elsewhere ^2,3^.

***Estimation methods***

We estimated the average effect of treatment on the treated (ATT) in both the simulated data and applied example, by applying seven matching approaches, three analysis methods, and two estimation approaches. The ATT estimand is the average difference between potential outcomes^4^ for the exposed units under exposure, and the exposed units had they been unexposed. The methods estimate the ATT by comparing the average outcome in the exposed group to the average outcome in a comparison group of unexposed units that has been selected, weighted, or otherwise adjusted to approximate the covariate distribution of the exposed units.

The matching and analysis methods, described in greater detail below, relied on estimation of the treatment mechanism, or propensity score ^5^, and the outcome model. We estimated these models in two ways: First, parametrically, by assuming a functional form (main terms only) and applying linear or logistic regression, and second, semi-parametrically, by applying the SuperLearner ensemble machine learning algorithm ^6–8^. While parametric approaches are standard and far more common in practice, recent evidence suggests that semi-parametric approaches may reduce bias and increase efficiency ^6,9,10^.

When analyzing the simulated data, we assumed parametric model forms that were misspecified given the data generating mechanism. This is because correct specification of the model form is unlikely in applied settings where the true underlying data generating mechanisms are unknown. Hence, the analysis aligns with what is done in practice. It further provides an opportunity to examine potential gains from semi-parametric estimation when the model form is not known.

***Matching methods***

Using the framing of Ho et al ^11^, we treated each matching procedure as a form of pre-processing, after which the ATT could be estimated by calculating the difference of mean outcomes between treated and controls units (a “naïve” analysis) or applying further analysis techniques. The matching approaches were: one-to-one greedy nearest neighbor (NN) matching with replacement ^12–15^; one-to-one optimal nearest neighbor (optimal) matching without replacement ^16–20^; subclassification using 10 quantiles in simulations and 5 quantiles in the applied example ^5,15,21–23^; full matching ^17,19,20,23,24^; inverse probability of treatment weighting (IPTW) ^5,25^; and genetic matching ^26,27^. We also considered unmatched data.

Each matching method is described in more detail below. Nearest neighbor matching utilizes the propensity score ^5^ to identify similarities between treated and control units. We estimated the propensity score in two ways, described in the *Estimation methods* section below. For greedy nearest neighbor matching, we selected a match for each treated unit one at a time, starting with the largest propensity score and progressing to the smallest. At each matching step, the control with the closest propensity score was selected in a greedy fashion. Controls were drawn with replacement, and those left un-matched were discarded.

For optimal nearest neighbor matching, units were matched 1:1 without replacement using an optimization routine designed to globally minimize the distances, measured by the propensity score, between treated and control units. The algorithm finds the smallest average absolute distance across all the matched pairs. Again, un-matched control units were dropped.

We conducted subclassification, also known as interval matching or blocking, by dividing the data into quantiles (10 in simulations and 5 in the applied example) of the propensity score distribution of the treated units, and assigning all controls to these groups based on the propensity score bounds. Comparisons of outcomes or further analyses were conducted within each subclass, and the overall estimate was the average of the subclass-specific estimates, weighted by the number of treated units in each subclass.

We used full matching to subclassify the data based on the propensity score in a globally optimal way. Each subclass had at least one treated unit and one control, with the number of subclasses selected as part of the optimization procedure, and all observations were retained. Comparison of outcomes or further analyses were done using the whole dataset where treated units receive a weight of 1 and controls receive a weight of 1 divided by the number of control units in the designated subclass.

Inverse probability of treatment weighting (IPTW) utilizes the propensity score to reweight the outcomes among exposed and unexposed units so that the two groups have the same covariate distributions.^5,25^ To estimate the ATT, all exposed units are assigned a weight of one and all unexposed units are assigned weights equal to the propensity odds (p/(1-p), where p is the propensity score for each unit). The weighting scheme aims to adjust the covariate distribution of unexposed units to match that of exposed units. IPTW weights can be applied in a naïve analysis or together with g-computation.

Genetic matching uses an iterative search algorithm to maximize the balance of potential covariates across treated and control observations by weighting each covariate. An initial set of weights is generated randomly and balance between treated and control groups is tested using paired t-tests for dichotomous variables and Kolmogorov-Smirnov tests for multinomial and continuous variables. Weights that achieved better balance are more likely to be retained, and others more likely to be dropped, and the retained weights are the basis for “mutations” to create a next generation of weights for testing. The new, mutated weights are applied, and balance is tested again; this process continues until no significant improvements in balance are observed for four consecutive generations. Matching was done without replacement, and some control units were dropped.

The matching methods relied on measures of the distance between covariate values in the treatment and control groups. In all cases except genetic matching, this distance metric was the propensity score, estimated parametrically and semi-parametrically, as described above. In the case of genetic matching, the distance measure was the generalized Mahalanobis metric, as recommended ^27^. Results for the parametric and semi-parametric matching approaches were very similar. Table S7 summarizes the median and range of the number of control units dropped, by data generating mechanism and matching method, for 50 simulated sample realizations and the applied example.

***Analysis methods***

Estimators of the ATT are available that adjust for covariates based on the treatment mechanism, the outcome mechanism, or both (also known as double-robust methods). After a matching approach, which utilizes the treatment mechanism, is applied, an analysis approach is used to compare outcomes in the matched samples. We considered three outcome analyses: a naïve analysis, g-computation^25^, and targeted minimum loss-based estimation (TMLE) ^28–30^. G-computation is a maximum likelihood based substitution estimator of the G-formula. It is implemented by using regression to model the outcome as a function of the exposure and relevant covariates. The fitted model is then used to predict the outcome under different counterfactual scenarios to be compared. To estimate the ATT, we average the difference between the model predictions for all exposed units had they been unexposed and the model predictions for all exposed units had they been exposed. Typically, G-computation relies on a parametric model. TMLE for the ATT is a general two-stage efficient substitution estimator. In the first stage, we model the outcome as a function of the exposure and relevant covariates. The second stage is a bias reduction step that iteratively updates the parameter estimates using models of the exposure given covariates (the treatment mechanism). This updating step also makes the estimator double-robust, asymptotically normal, and asymptotically efficient. TMLE is typically implemented with semi-parametric machine learning methods. Treatment and outcome models for TMLE were estimated using parametric and semi-parametric approaches, as described above. For g-computation, we used only parametric estimation, as inference using semi-parametric g-computation has not been established.

***Balance and performance metrics***

For each matched sample we calculated a large set of balance metrics recommended in the literature ^11,27,31–34^. These included: the mean, median, and maximum absolute standardized mean difference (ASMD) in covariate values between the treated and control groups across all eight covariates, the percent of covariates with ASMD less than 20%, 10%, 5%, and 1%, the ASMD of the prognostic score, the ASMD of the propensity score, and the absolute mean t-statistic comparing covariate values between treatment and control group.

The propensity scores and prognostic scores used to measure balance were distinct from those used to estimate effects. In the simulated data, the propensity score and prognostic score were quantified using generalized linear regression and the known treatment and outcome model forms (see above). In the NSW data, the propensity score and prognostic score were estimated using main terms logistic regression and SuperLearner. In the latter case (the applied example), the propensity scores and prognostic scores were indistinguishable across the two estimation procedures, so only the former (logistic regresion) is reported. We also present plots of the distributions of propensity scores by treated and control groups to illustrate the degree of covariate support in the different scenarios ^5,35^.

For each data generating mechanism, we created 1,000 simulated datasets and evaluated the performance of each matching and analysis combination. The primary measure of performance was the mean squared error (MSE = bias² + variance), where lower MSE indicates a better estimate. We also compared the average percent bias and the variance. We present all three performance measures for each of the estimators considered. To estimate the bias, variance, and MSE of the effect estimates in the applied example and to account for stochastic elements of the matching and machine learning algorithms, we analyzed 500 bootstrapped datasets ^23,31,36–38^.

All analyses were conducted in R version 3.2.0 ^39^. Matching was implemented using the MatchIt package ^40^, and its dependent packages. TMLE was implemented using a modified (to incorporate matching weights) version of tmlecte package available on Github^41^.

**Additional results**

Simulation results for all tested matching, analysis, and estimation approaches for the good, medium, and poor support scenarios are presented in Tables S1-S3. Balance for all parametric matching methods, all simulation scenarios, and all metrics are presented in Table S4.

Balance for all parametric matching methods and metrics in the applied example are presented in Table S5

***Simulation results for the medium support scenario***

The probability of treatment, also known as the propensity score, is a useful metric for examining covariate support ^5,35^. The distributions of the propensity score, by treated and control units, are presented for the medium support scenario in Fig. S1. The probability of treatment ranged between 0.003 and 0.984.

In the medium support scenario (Table S2), several matching and analysis methods performed well, though fewer than in the good support scenario. Across the 52 methods, 29% had MSE less than 0.01 and 48% were less than 1% biased. In terms of MSE, semi-parametric optimal matching with semi-parametric TMLE performed best, and the top ten performers all involved TMLE. The benefits of combining matching with subsequent analysis are more apparent in this scenario, with fewer high-performing methods involving matching alone. Among the matching methods, optimal matching with no subsequent analysis was one of worst-performing methods, however optimal matching with semi-parametric TMLE was the best-performing method, highlighting how matching can be substantially improved by further analysis. The lowest bias was achieved with semi-parametric subclassification and g-computation.

**
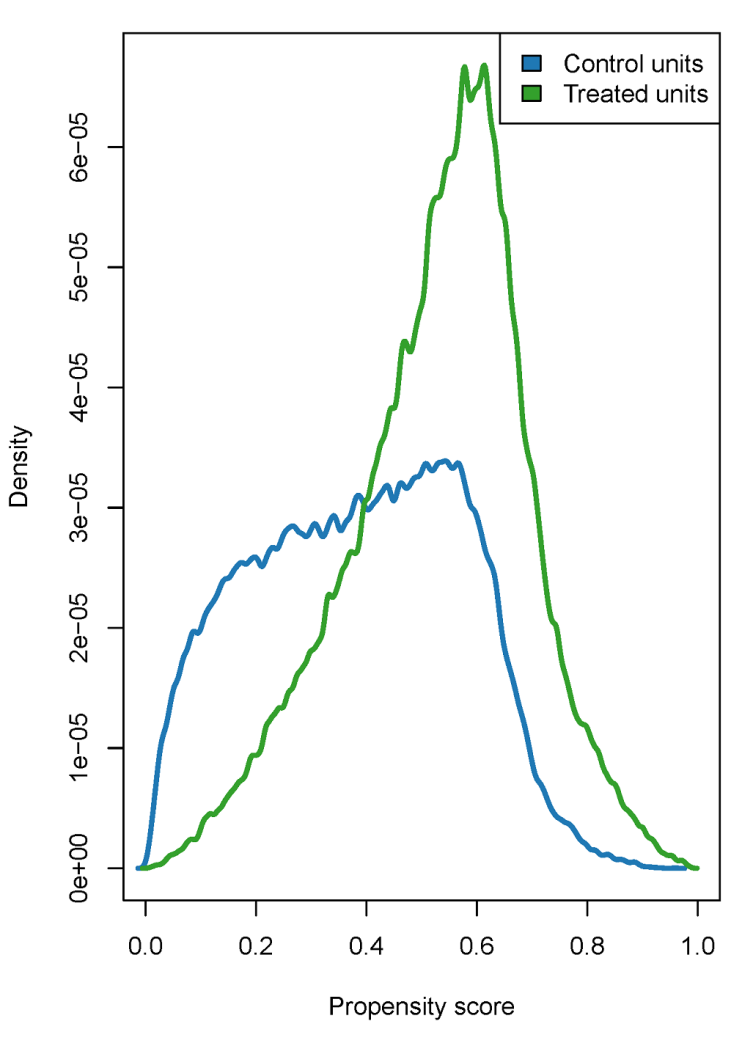
**

**Fig. S1.** Density of estimated propensity scores for treated and control units in medium support scenario

Table S1. Simulation results comparing matching and analysis combinations in good support scenario

| **Match** | **Analysis** | **% Bias** | **Var** | **MSE** | **Bias rank** | **Var rank** | **MSE rank** |
| --- | --- | --- | --- | --- | --- | --- | --- |
| Full | TMLE parametric | 0.28% | 0.0063 | 0.0063 | 27 | 2 | 1 |
| Full + SL | TMLE parametric | 0.34% | 0.0063 | 0.0064 | 30 | 3 | 2 |
| None | TMLE with SL | -0.02% | 0.0065 | 0.0065 | 8 | 4 | 3 |
| None | TMLE parametric | -0.14% | 0.0066 | 0.0066 | 19 | 5 | 4 |
| IPTW | G-computation | 0.13% | 0.0066 | 0.0066 | 16 | 6 | 5 |
| IPTW + SL | TMLE with SL | -0.02% | 0.0066 | 0.0066 | 7 | 8 | 6 |
| IPTW | TMLE with SL | 0.02% | 0.0067 | 0.0067 | 6 | 10 | 7 |
| Sub + SL | G-computation | -0.32% | 0.0067 | 0.0067 | 28 | 11 | 8 |
| IPTW + SL | G-computation | -0.76% | 0.0066 | 0.0067 | 37 | 7 | 9 |
| Sub | G-computation | -0.48% | 0.0067 | 0.0067 | 33 | 12 | 10 |
| IPTW | Naïve | -0.52% | 0.0068 | 0.0069 | 34 | 15 | 11 |
| Sub | TMLE with SL | -0.45% | 0.0069 | 0.0070 | 32 | 16 | 12 |
| IPTW + SL | Naïve | 1.40% | 0.0067 | 0.0071 | 43 | 9 | 13 |
| Opt | G-computation | -1.35% | 0.0067 | 0.0071 | 42 | 13 | 14 |
| Full + SL | TMLE with SL | 0.00% | 0.0071 | 0.0071 | 2 | 19 | 15 |
| Sub + SL | Naïve | 0.77% | 0.0070 | 0.0071 | 38 | 18 | 16 |
| Sub | Naïve | 0.89% | 0.0070 | 0.0072 | 40 | 17 | 17 |
| Opt + SL | G-computation | -1.41% | 0.0068 | 0.0072 | 44 | 14 | 18 |
| Opt | TMLE with SL | -0.08% | 0.0072 | 0.0072 | 11 | 22 | 19 |
| Full | TMLE with SL | -0.04% | 0.0073 | 0.0073 | 9 | 23 | 20 |
| Opt + SL | TMLE parametric | -0.86% | 0.0072 | 0.0073 | 39 | 21 | 21 |
| Opt + SL | TMLE with SL | 0.00% | 0.0073 | 0.0073 | 1 | 24 | 22 |
| Opt | TMLE parametric | -1.25% | 0.0071 | 0.0074 | 41 | 20 | 23 |
| Full + SL | G-computation | 0.02% | 0.0076 | 0.0076 | 3 | 25 | 24 |
| Full | G-computation | -0.21% | 0.0077 | 0.0077 | 23 | 27 | 25 |
| Sub + SL | TMLE with SL | -0.55% | 0.0078 | 0.0078 | 35 | 28 | 26 |
| Full + SL | Naïve | -0.02% | 0.0079 | 0.0079 | 4 | 29 | 27 |
| Full | Naïve | 0.24% | 0.0080 | 0.0081 | 25 | 30 | 28 |
| Sub | TMLE parametric | -1.52% | 0.0082 | 0.0087 | 46 | 32 | 29 |
| Genetic | TMLE with SL | 0.11% | 0.0090 | 0.0090 | 13 | 34 | 30 |
| Sub + SL | TMLE parametric | -1.45% | 0.0086 | 0.0090 | 45 | 33 | 31 |
| Genetic + SL | TMLE with SL | 0.13% | 0.0092 | 0.0092 | 15 | 35 | 32 |
| Genetic | G-computation | 0.05% | 0.0093 | 0.0093 | 10 | 36 | 33 |
| Genetic | Naïve | 0.14% | 0.0093 | 0.0093 | 18 | 37 | 34 |
| Genetic + SL | G-computation | 0.09% | 0.0095 | 0.0095 | 12 | 38 | 35 |
| Genetic + SL | Naïve | 0.16% | 0.0095 | 0.0095 | 20 | 39 | 36 |
| NN + SL | TMLE with SL | 0.19% | 0.0097 | 0.0097 | 21 | 40 | 37 |
| NN | TMLE with SL | 0.02% | 0.0102 | 0.0102 | 5 | 43 | 38 |
| NN + SL | G-computation | 0.19% | 0.0103 | 0.0103 | 22 | 44 | 39 |
| Genetic + SL | TMLE parametric | 0.44% | 0.0105 | 0.0105 | 31 | 45 | 40 |
| Opt | Naïve | 2.23% | 0.0097 | 0.0107 | 47 | 41 | 41 |
| NN | G-computation | -0.23% | 0.0107 | 0.0107 | 24 | 46 | 42 |
| NN + SL | Naïve | 0.14% | 0.0108 | 0.0108 | 17 | 47 | 43 |
| NN + SL | TMLE parametric | 0.55% | 0.0110 | 0.0111 | 36 | 48 | 44 |
| NN | Naïve | 0.28% | 0.0113 | 0.0113 | 26 | 49 | 45 |
| Opt + SL | Naïve | 2.63% | 0.0100 | 0.0114 | 48 | 42 | 46 |
| Genetic | TMLE parametric | 0.33% | 0.0115 | 0.0115 | 29 | 50 | 47 |
| NN | TMLE parametric | -0.12% | 0.0129 | 0.0129 | 14 | 51 | 48 |
| None | G-computation | -8.51% | 0.0054 | 0.0202 | 51 | 1 | 49 |
| IPTW | TMLE parametric | 7.97% | 0.0077 | 0.0207 | 49 | 26 | 50 |
| IPTW + SL | TMLE parametric | 8.29% | 0.0082 | 0.0223 | 50 | 31 | 51 |
| None | Naïve | 21.22% | 0.0147 | 0.1073 | 52 | 52 | 52 |

Var: variance. MSE: mean squared error. NN: greedy nearest neighbor. Opt: optimal nearest neighbor. Sub: subclassification. IPTW: inverse probability of treatment weighting. SL: using SuperLearner for semi-parametric estimation. TMLE: targeted minimum loss-based estimation.

**Table S2**. Simulation results comparing matching and analysis combinations in medium support scenario

| **Match** | **Analysis** | **% Bias** | **Var** | **MSE** | **Bias rank** | **Var rank** | **MSE rank** |
| --- | --- | --- | --- | --- | --- | --- | --- |
| Opt + SL | TMLE with SL | 0.82% | 0.0081 | 0.0082 | 19 | 4 | 1 |
| Opt + SL | TMLE parametric | -0.51% | 0.0082 | 0.0082 | 4 | 8 | 2 |
| Full + SL | TMLE with SL | 0.56% | 0.0082 | 0.0083 | 6 | 9 | 3 |
| Opt | TMLE with SL | 0.80% | 0.0081 | 0.0083 | 17 | 5 | 4 |
| None | TMLE parametric | -0.80% | 0.0082 | 0.0083 | 18 | 7 | 5 |
| Opt | TMLE parametric | -1.00% | 0.0081 | 0.0084 | 26 | 6 | 6 |
| IPTW | TMLE with SL | 0.73% | 0.0083 | 0.0084 | 15 | 10 | 7 |
| Full | TMLE with SL | 0.60% | 0.0084 | 0.0085 | 9 | 11 | 8 |
| IPTW + SL | TMLE with SL | 0.71% | 0.0084 | 0.0085 | 13 | 12 | 9 |
| None | TMLE with SL | 0.86% | 0.0084 | 0.0086 | 21 | 13 | 10 |
| Sub | G-computation | -1.09% | 0.0084 | 0.0087 | 27 | 14 | 11 |
| Sub + SL | G-computation | 0.04% | 0.0089 | 0.0089 | 1 | 18 | 12 |
| IPTW + SL | G-computation | 0.41% | 0.0091 | 0.0091 | 2 | 21 | 13 |
| Sub | TMLE with SL | -1.14% | 0.0090 | 0.0094 | 28 | 20 | 14 |
| IPTW | G-computation | 1.59% | 0.0090 | 0.0096 | 31 | 19 | 15 |
| Genetic + SL | TMLE with SL | 0.72% | 0.0102 | 0.0104 | 14 | 26 | 16 |
| IPTW + SL | Naïve | 1.47% | 0.0099 | 0.0105 | 30 | 25 | 17 |
| Genetic | TMLE with SL | 0.85% | 0.0104 | 0.0106 | 20 | 27 | 18 |
| IPTW | TMLE parametric | -2.38% | 0.0093 | 0.0108 | 33 | 24 | 19 |
| Full + SL | G-computation | 0.57% | 0.0107 | 0.0108 | 7 | 30 | 20 |
| Full | G-computation | -0.43% | 0.0108 | 0.0109 | 3 | 32 | 21 |
| Sub + SL | TMLE with SL | -0.90% | 0.0107 | 0.0109 | 22 | 29 | 22 |
| NN + SL | TMLE with SL | 0.70% | 0.0108 | 0.0109 | 12 | 31 | 23 |
| Sub + SL | Naïve | 2.64% | 0.0092 | 0.0109 | 36 | 22 | 24 |
| Full | TMLE parametric | -3.03% | 0.0086 | 0.0110 | 38 | 15 | 25 |
| Genetic + SL | G-computation | 0.56% | 0.0113 | 0.0113 | 5 | 34 | 26 |
| Genetic | G-computation | 0.64% | 0.0113 | 0.0114 | 10 | 35 | 27 |
| NN | TMLE with SL | 0.77% | 0.0113 | 0.0115 | 16 | 37 | 28 |
| Genetic + SL | Naïve | 0.94% | 0.0113 | 0.0116 | 24 | 38 | 29 |
| Full + SL | TMLE parametric | -3.30% | 0.0088 | 0.0116 | 39 | 16 | 30 |
| IPTW | Naïve | -1.98% | 0.0106 | 0.0116 | 32 | 28 | 31 |
| Full + SL | Naïve | 0.91% | 0.0114 | 0.0116 | 23 | 39 | 32 |
| Genetic | Naïve | 1.23% | 0.0113 | 0.0117 | 29 | 36 | 33 |
| NN + SL | G-computation | 0.57% | 0.0127 | 0.0128 | 8 | 41 | 34 |
| Full | Naïve | 2.57% | 0.0111 | 0.0128 | 35 | 33 | 35 |
| NN | G-computation | -0.68% | 0.0128 | 0.0129 | 11 | 42 | 36 |
| Sub | Naïve | 4.05% | 0.0088 | 0.0130 | 40 | 17 | 37 |
| NN + SL | Naïve | 0.98% | 0.0131 | 0.0133 | 25 | 43 | 38 |
| IPTW + SL | TMLE parametric | -4.10% | 0.0093 | 0.0136 | 41 | 23 | 39 |
| NN | Naïve | 2.56% | 0.0132 | 0.0148 | 34 | 44 | 40 |
| Sub + SL | TMLE parametric | -2.75% | 0.0154 | 0.0173 | 37 | 46 | 41 |
| Sub | TMLE parametric | -5.36% | 0.0125 | 0.0198 | 43 | 40 | 42 |
| NN | TMLE parametric | -6.10% | 0.0184 | 0.0279 | 45 | 49 | 43 |
| Genetic | TMLE parametric | -6.83% | 0.0236 | 0.0355 | 46 | 50 | 44 |
| Opt | G-computation | -13.95% | 0.0076 | 0.0576 | 47 | 3 | 45 |
| Opt + SL | G-computation | -14.41% | 0.0074 | 0.0608 | 48 | 2 | 46 |
| NN + SL | TMLE parametric | -5.14% | 0.0596 | 0.0663 | 42 | 52 | 47 |
| Genetic + SL | TMLE parametric | -5.72% | 0.0590 | 0.0674 | 44 | 51 | 48 |
| None | G-computation | -16.86% | 0.0067 | 0.0798 | 49 | 1 | 49 |
| Opt | Naïve | 25.57% | 0.0167 | 0.1847 | 50 | 48 | 50 |
| Opt + SL | Naïve | 27.33% | 0.0163 | 0.2082 | 51 | 47 | 51 |
| None | Naïve | 40.95% | 0.0138 | 0.4446 | 52 | 45 | 52 |

Var: variance. MSE: mean squared error. NN: greedy nearest neighbor. Opt: optimal nearest neighbor. Sub: subclassification. IPTW: inverse probability of treatment weighting. SL: using SuperLearner for semi-parametric estimation. TMLE: targeted minimum loss-based estimation.

Table S3. Simulation results comparing matching and analysis combinations in poor support scenario

| **Match** | **Analysis** | **% Bias** | **Var** | **MSE** | **Bias rank** | **Var rank** | **MSE rank** |
| --- | --- | --- | --- | --- | --- | --- | --- |
| Full + SL | TMLE with SL | -0.82% | 0.0088 | 0.0090 | 17 | 4 | 1 |
| Full | TMLE with SL | -0.81% | 0.0088 | 0.0091 | 15 | 5 | 2 |
| IPTW | TMLE with SL | -0.76% | 0.0090 | 0.0092 | 13 | 6 | 3 |
| IPTW + SL | TMLE with SL | -0.73% | 0.0091 | 0.0092 | 11 | 7 | 4 |
| Genetic | TMLE with SL | -0.65% | 0.0130 | 0.0132 | 7 | 14 | 5 |
| Genetic + SL | TMLE with SL | -0.81% | 0.0130 | 0.0132 | 16 | 13 | 6 |
| NN + SL | TMLE with SL | -0.75% | 0.0134 | 0.0136 | 12 | 15 | 7 |
| NN | TMLE with SL | -0.70% | 0.0140 | 0.0141 | 9 | 16 | 8 |
| Opt + SL | TMLE with SL | 0.48% | 0.0165 | 0.0165 | 3 | 24 | 9 |
| Opt | TMLE with SL | 0.53% | 0.0166 | 0.0167 | 6 | 25 | 10 |
| IPTW | G-computation | -0.70% | 0.0169 | 0.0171 | 10 | 26 | 11 |
| None | TMLE with SL | 0.52% | 0.0179 | 0.0179 | 5 | 27 | 12 |
| IPTW + SL | G-computation | -1.22% | 0.0188 | 0.0193 | 20 | 28 | 13 |
| Sub | Naïve | 3.47% | 0.0158 | 0.0201 | 33 | 23 | 14 |
| Sub + SL | Naïve | 2.50% | 0.0189 | 0.0210 | 31 | 29 | 15 |
| Genetic | G-computation | -0.78% | 0.0210 | 0.0212 | 14 | 32 | 16 |
| Full | G-computation | -1.55% | 0.0204 | 0.0212 | 23 | 30 | 17 |
| Full | Naïve | 1.77% | 0.0210 | 0.0221 | 27 | 31 | 18 |
| Genetic | Naïve | 1.59% | 0.0216 | 0.0224 | 25 | 33 | 19 |
| Genetic + SL | G-computation | -0.67% | 0.0226 | 0.0228 | 8 | 36 | 20 |
| NN | G-computation | -1.82% | 0.0217 | 0.0229 | 28 | 34 | 21 |
| NN | Naïve | 1.76% | 0.0222 | 0.0232 | 26 | 35 | 22 |
| Full + SL | G-computation | -0.29% | 0.0235 | 0.0235 | 1 | 38 | 23 |
| Genetic + SL | Naïve | 1.51% | 0.0233 | 0.0241 | 22 | 37 | 24 |
| NN + SL | G-computation | -0.49% | 0.0245 | 0.0246 | 4 | 40 | 25 |
| Opt | TMLE parametric | 1.11% | 0.0247 | 0.0251 | 18 | 41 | 26 |
| Opt + SL | TMLE parametric | 1.57% | 0.0248 | 0.0256 | 24 | 42 | 27 |
| Full + SL | Naïve | 1.24% | 0.0252 | 0.0257 | 21 | 43 | 28 |
| NN + SL | Naïve | 1.17% | 0.0260 | 0.0265 | 19 | 44 | 29 |
| Sub | G-computation | -3.46% | 0.0243 | 0.0285 | 32 | 39 | 30 |
| None | TMLE parametric | 1.89% | 0.0285 | 0.0297 | 29 | 45 | 31 |
| Genetic | TMLE parametric | -6.69% | 0.0145 | 0.0303 | 36 | 19 | 32 |
| Genetic + SL | TMLE parametric | -6.76% | 0.0142 | 0.0303 | 37 | 17 | 33 |
| NN + SL | TMLE parametric | -6.62% | 0.0153 | 0.0308 | 35 | 22 | 34 |
| IPTW | TMLE parametric | -7.56% | 0.0106 | 0.0308 | 40 | 9 | 35 |
| IPTW + SL | TMLE parametric | -7.62% | 0.0105 | 0.0310 | 41 | 8 | 36 |
| NN | TMLE parametric | -7.22% | 0.0147 | 0.0331 | 38 | 21 | 37 |
| Full | TMLE parametric | -7.84% | 0.0118 | 0.0335 | 43 | 11 | 38 |
| Full + SL | TMLE parametric | -7.94% | 0.0119 | 0.0341 | 44 | 12 | 39 |
| Sub + SL | G-computation | -4.81% | 0.0356 | 0.0437 | 34 | 46 | 40 |
| IPTW | Naïve | -1.94% | 0.0493 | 0.0506 | 30 | 49 | 41 |
| Sub | TMLE parametric | -7.50% | 0.0467 | 0.0665 | 39 | 47 | 42 |
| Sub + SL | TMLE parametric | -7.76% | 0.0487 | 0.0699 | 42 | 48 | 43 |
| Sub | TMLE with SL | -8.04% | 0.0512 | 0.0740 | 45 | 50 | 44 |
| Sub + SL | TMLE with SL | -10.08% | 0.0587 | 0.0945 | 46 | 51 | 45 |
| IPTW + SL | Naïve | -0.29% | 0.1033 | 0.1032 | 2 | 52 | 46 |
| None | G-computation | -26.75% | 0.0074 | 0.2605 | 47 | 1 | 47 |
| Opt + SL | G-computation | -28.50% | 0.0074 | 0.2947 | 48 | 2 | 48 |
| Opt | G-computation | -28.68% | 0.0074 | 0.2982 | 49 | 3 | 49 |
| Opt | Naïve | 45.27% | 0.0147 | 0.7393 | 50 | 20 | 50 |
| Opt + SL | Naïve | 46.15% | 0.0145 | 0.7676 | 51 | 18 | 51 |
| None | Naïve | 60.41% | 0.0110 | 1.3013 | 52 | 10 | 52 |

Var: variance. MSE: mean squared error. NN: greedy nearest neighbor. Opt: optimal nearest neighbor. Sub: subclassification. IPTW: inverse probability of treatment weighting. SL: using SuperLearner for semi-parametric estimation. TMLE: targeted minimum loss-based estimation.

**Table S4: Balance metrics by simulation scenario and matching method**

| **Match method** | **Percent of covariates with ASMD less than…** | | | | **Mean ASMD** | **Median ASMD** | **Max. ASMD** | **ASMD in prog.**  **Score** | **ASMD in propen. score** | **Abs mean**  **t-stat** |
| --- | --- | --- | --- | --- | --- | --- | --- | --- | --- | --- |
|  | **20%** | **10%** | **5%** | **1%** |  |  |  |  |  |  |
| **Good support** | | | | | | | | | | |
| None | 46.1 | 15.8 | 5.3 | 0.9 | 0.216 | 0.214 | 0.443 | 0.301 | 0.362 | 3.360 |
| NN | 100 | 96.5 | 80.6 | 29.9 | 0.031 | 0.024 | 0.147 | 0.020 | 0.072 | 0.403 |
| Opt | 100 | 97.5 | 81.1 | 23.5 | 0.030 | 0.025 | 0.148 | 0.039 | 0.069 | 0.446 |
| Genetic | **100** | **100** | **100** | **98.8** | **0.001** | **0.000** | **0.024** | **0.003** | **0.006** | **0.018** |
| Sub | 100 | 100 | 99.7 | 41.7 | 0.014 | 0.013 | 0.055 | 0.018 | 0.048 | 0.221 |
| Full | 100 | 99.9 | 94.7 | 39.9 | 0.018 | 0.014 | 0.092 | 0.013 | 0.054 | 0.280 |
| IPTW | 100 | 100 | 99.7 | 42.6 | 0.015 | 0.013 | 0.072 | 0.013 | 0.068 | 0.212 |
| **Medium support** | | | | | | | | | | |
| None | 0.5 | 0.0 | 0.0 | 0.0 | 0.507 | 0.505 | 0.724 | 0.675 | 0.776 | 7.957 |
| NN | 100 | 89.9 | 59.7 | 11.1 | 0.044 | 0.038 | 0.164 | 0.039 | 0.092 | 0.565 |
| Opt | 24.8 | 2.5 | 0.1 | 0.0 | 0.304 | 0.299 | 0.595 | 0.410 | 0.430 | 4.805 |
| Genetic | **100** | **100** | **99.5** | **75.8** | **0.007** | **0.005** | **0.057** | **0.009** | **0.010** | **0.090** |
| Sub | 100 | 98.0 | 47.3 | 4.6 | 0.051 | 0.050 | 0.136 | 0.059 | 0.107 | 0.819 |
| Full | 100 | 92.6 | 62.6 | 11.1 | 0.046 | 0.042 | 0.186 | 0.038 | 0.108 | 0.734 |
| IPTW | 99.6 | 69.8 | 30.0 | 5.9 | 0.052 | 0.048 | 0.245 | 0.060 | 0.157 | 0.791 |
| **Poor support** | | | | | | | | | | |
| None | 48.1 | 28.9 | 13.8 | 2.8 | 0.728 | 0.726 | 0.941 | 1.347 | 1.184 | 11.446 |
| NN | 74.4 | 57.3 | 36.5 | 5.2 | 0.097 | 0.090 | 0.330 | 0.075 | 0.126 | 1.069 |
| Opt | 49.6 | 39.5 | 20.7 | 5.0 | 0.548 | 0.544 | 0.822 | 1.020 | 0.953 | 9.155 |
| Genetic | **99.1** | **88.1** | **64.6** | **12.9** | **0.049** | **0.042** | **0.220** | **0.047** | **0.038** | **0.574** |
| Sub | 78.9 | 39.0 | 7.4 | 1.0 | 0.137 | 0.131 | 0.451 | 0.106 | 0.132 | 2.162 |
| Full | 79.0 | 58.1 | 36.3 | 3.5 | 0.114 | 0.111 | 0.335 | 0.074 | 0.145 | 1.800 |
| IPTW | 62.5 | 30.0 | 15.1 | 2.7 | 0.161 | 0.130 | 1.643 | 0.154 | 0.200 | 2.245 |

ASMD: Absolute standardized mean difference in covariate values between treated and control groups. Max: maximum. Prog: prognostic. Propen: propensity. Abs: absolute. Stat: statistic. NN: greedy nearest neighbor. Opt: optimal nearest neighbor. Sub: subclassification. IPTW: Inverse probability of treatment weighting. Metrics are averaged across 1,000 simulation runs. Bolded values indicate the best balance according to each metric and scenario.

**Table S5: Balance metrics for NSW observational data by matching method**

| **Match method** | **Percent of covariates with ASMD less than…** | | | | **Mean ASMD** | **Median ASMD** | **Max. ASMD** | **ASMD in prog.**  **score** | **ASMD in propen. score** | **Abs mean**  **t-stat** |
| --- | --- | --- | --- | --- | --- | --- | --- | --- | --- | --- |
|  | **20%** | **10%** | **5%** | **1%** |  |  |  |  |  |  |
| None | 12.2 | 9.3 | 5.0 | 0.8 | 1.184 | 1.182 | 1.471 | 1.384 | 4.901 | 21.371 |
| NN | 94.4 | 71.8 | 45.1 | 8.5 | 0.076 | 0.066 | 0.309 | 0.044 | 0.490 | 0.737 |
| Opt | 98.6 | 83.3 | 56.2 | 11.8 | 0.055 | 0.048 | 0.227 | 0.028 | 0.492 | 0.615 |
| Genetic | **100** | 98.8 | **93.5** | **60.5** | **0.014** | **0.008** | 0.160 | 0.013 | 0.060 | **0.160** |
| Sub | 60.0 | 16.5 | 4.8 | 0.8 | 0.182 | 0.181 | 0.352 | 0.234 | 0.062 | 3.454 |
| Full | 97.9 | 83.4 | 58.3 | 14.6 | 0.055 | 0.048 | 0.215 | 0.024 | **0.012** | 0.902 |
| IPTW | 100 | **99.8** | 91.8 | 54.5 | 0.016 | 0.015 | **0.048** | **0.013** | 0.028 | 0.172 |

ASMD: Absolute standardized mean difference in covariate values between treated and control groups. Max: maximum. Prog: prognostic. Propen: propensity. Abs: absolute. Stat: statistic. NN: greedy nearest neighbor. Opt: optimal nearest neighbor. Sub: subclassification. IPTW: Inverse probability of treatment weighting. Metrics are averaged across 1,000 simulation runs. Bolded values indicate the best balance according to each metric and scenario.

Table S6. Results comparing matching and analysis combinations for NSW observational data

| **Match** | **Analysis** | **% Bias** | **Var** | **MSE** | **Bias rank** | **Var rank** | **MSE rank** |
| --- | --- | --- | --- | --- | --- | --- | --- |
| Full + SL | TMLE with SL | -19.04% | 415857 | 531741 | 22 | 3 | 1 |
| Full | TMLE with SL | -19.19% | 422780 | 540433 | 23 | 6 | 2 |
| None | TMLE with SL | -21.60% | 452307 | 601601 | 26 | 12 | 3 |
| IPTW | TMLE with SL | -23.29% | 450296 | 624041 | 28 | 10 | 4 |
| Opt + SL | TMLE with SL | 0.50% | 649554 | 648337 | 2 | 28 | 5 |
| Genetic + SL | TMLE with SL | -5.43% | 647301 | 655516 | 8 | 27 | 6 |
| Opt + SL | TMLE parametric | -17.96% | 561525 | 664304 | 20 | 21 | 7 |
| Genetic | TMLE with SL | -13.85% | 612929 | 673484 | 14 | 26 | 8 |
| Opt | TMLE with SL | -2.21% | 687593 | 687788 | 4 | 29 | 9 |
| IPTW + SL | G-computation | -27.89% | 443257 | 692890 | 34 | 9 | 10 |
| Full | G-computation | -23.22% | 521981 | 694515 | 27 | 16 | 11 |
| IPTW + SL | TMLE with SL | -24.30% | 505967 | 695003 | 30 | 14 | 12 |
| Opt | TMLE parametric | -23.88% | 522234 | 704868 | 29 | 17 | 13 |
| Genetic | G-computation | -0.78% | 716401 | 715161 | 3 | 30 | 14 |
| Opt + SL | Naïve | -20.35% | 585512 | 717624 | 25 | 24 | 15 |
| None | TMLE parametric | -30.34% | 423477 | 718995 | 38 | 7 | 16 |
| Genetic | Naïve | -2.78% | 719467 | 720521 | 5 | 32 | 17 |
| Full + SL | G-computation | -18.78% | 610874 | 723196 | 21 | 25 | 18 |
| Opt | G-computation | -25.40% | 517669 | 724374 | 32 | 15 | 19 |
| Full + SL | Naïve | -8.63% | 716612 | 739178 | 11 | 31 | 20 |
| Opt + SL | G-computation | -25.05% | 550151 | 751031 | 31 | 19 | 21 |
| IPTW + SL | Naïve | -30.55% | 456890 | 756393 | 39 | 13 | 22 |
| IPTW | G-computation | -33.71% | 420389 | 785451 | 40 | 4 | 23 |
| Full | Naïve | -26.56% | 562168 | 788141 | 33 | 22 | 24 |
| IPTW | Naïve | -35.10% | 427309 | 823093 | 41 | 8 | 25 |
| Genetic | TMLE parametric | -16.40% | 746049 | 831166 | 18 | 33 | 26 |
| Opt | Naïve | -29.63% | 555443 | 837056 | 36 | 20 | 27 |
| Sub | G-computation | -29.52% | 565009 | 844370 | 35 | 23 | 28 |
| Genetic + SL | G-computation | 13.76% | 823567 | 882869 | 13 | 35 | 29 |
| NN | G-computation | -14.66% | 815658 | 883232 | 15 | 34 | 30 |
| Genetic + SL | Naïve | 14.80% | 830376 | 899270 | 16 | 37 | 31 |
| NN | Naïve | -16.23% | 825951 | 909128 | 17 | 36 | 32 |
| NN + SL | Naïve | 0.28% | 928682 | 926849 | 1 | 41 | 33 |
| NN + SL | TMLE with SL | -8.34% | 911983 | 932539 | 10 | 40 | 34 |
| NN | TMLE parametric | -17.23% | 838994 | 932869 | 19 | 38 | 35 |
| NN + SL | G-computation | -9.64% | 911617 | 939693 | 12 | 39 | 36 |
| Genetic + SL | TMLE parametric | -7.40% | 936134 | 951889 | 9 | 42 | 37 |
| NN | TMLE with SL | 3.57% | 1014406 | 1016483 | 6 | 43 | 38 |
| Full + SL | TMLE parametric | -48.24% | 420902 | 1169232 | 44 | 5 | 39 |
| Sub + SL | G-computation | -19.53% | 1069466 | 1190175 | 24 | 44 | 40 |
| None | G-computation | -62.00% | 371377 | 1608178 | 46 | 2 | 41 |
| Sub | TMLE with SL | -40.19% | 1464808 | 1981612 | 42 | 45 | 42 |
| Sub + SL | Naïve | -91.35% | 546518 | 3232076 | 48 | 18 | 43 |
| Sub | Naïve | -115.05% | 450462 | 4711305 | 50 | 11 | 44 |
| IPTW | TMLE parametric | -58.03% | 5026602 | 6100685 | 45 | 46 | 45 |
| Sub + SL | TMLE with SL | -64.54% | 5033750 | 6362569 | 47 | 47 | 46 |
| NN + SL | TMLE parametric | -5.40% | 7810570 | 7804329 | 7 | 50 | 47 |
| Full | TMLE parametric | -42.23% | 7325018 | 7884395 | 43 | 49 | 48 |
| Sub | TMLE parametric | -104.42% | 6304908 | 9790349 | 49 | 48 | 49 |
| IPTW + SL | TMLE parametric | -30.21% | 10069490 | 10342520 | 37 | 51 | 50 |
| Sub + SL | TMLE parametric | -155.36% | 16833984 | 24528132 | 51 | 52 | 51 |
| None | Naïve | -575.16% | 362337 | 106871723 | 52 | 1 | 52 |

Var: variance. MSE: mean squared error. NN: greedy nearest neighbor. Opt: optimal nearest neighbor. Sub: subclassification. IPTW: inverse probability of treatment weighting. SL: using SuperLearner for semi-parametric estimation. TMLE: targeted minimum loss-based estimation.

Table S7. Number of control units dropped by data generating mechanism and matching method

| **Matching method** | **Estimation** | **DGM 1**  (median [range]) | **DGM 2**  (median [range]) | **DGM 3**  (median [range]) | **Applied example**  (number) |
| --- | --- | --- | --- | --- | --- |
| None | N/A | 0 (0 – 0) | 0 (0 – 0) | 0 (0 – 0) | 0 |
| Greedy nearest neighbor | Parametric | 337.5  (290 – 378) | 311  (263 – 350) | 378  (324 – 419) | 15,860 |
| Greedy nearest neighbor | Semi-parametric | 337  (287 – 389) | 312.5  (278 – 361) | 378.5  (334 – 423) | 15,872 |
| Optimal nearest neighbor | Parametric | 184  (104 – 250) | 91  (38 – 176) | 109  (40 – 180) | 15,807 |
| Optimal nearest neighbor | Semi-parametric | 184  (104 – 250) | 91  (38 – 176) | 109  (40 – 180) | 15,807 |
| Subclassification | Parametric | 0 (0 – 0) | 0 (0 – 0) | 0 (0 – 0) | 0 |
| Subclassification | Semi-parametric | 0 (0 – 0) | 0 (0 – 0) | 0 (0 – 0) | 0 |
| IPTW | Parametric | 0 (0 – 0) | 0 (0 – 0) | 0 (0 – 0) | 0 |
| IPTW | Semi-parametric | 0 (0 – 0) | 0 (0 – 0) | 0 (0 – 0) | 0 |
| Genetic | Parametric | 322.5  (272 – 367) | 300.5  (259 – 344) | 372.5  (323 – 422) | 15,848 |
| Genetic | Semi-parametric | 325  (273 – 361) | 303  (269 – 344) | 375  (320 – 428) | 15,859 |
| Number of control units | | 592  (552 – 625) | 545  (519 – 588) | 554  (520 - 590) | 15,992 |
| Number of treated units | | 408  (375 – 448) | 455  (412 – 481) | 446  (410 – 480) | 185 |

Median and range of number of control units dropped, by data generating mechanism and matching method, for 50 simulated sample realizations and the applied example.

**References**

1. Imbens, G. W. Nonparametric estimation of average treatment effects under exogeneity: a review. *Rev. Econ. Stat.* **86,** 4–29 (2004).

2. LaLonde, R. J. Evaluating the econometric evaluations of training programs with experimental data. *Am. Econ. Rev.* **76,** 604–620 (1986).

3. Dehejia, R. H. & Wahba, S. Causal effects in nonexperimental studies: reevaluating the evaluation of training programs. *J. Am. Stat. Assoc.* **94,** 1053–1062 (1999).

4. Rubin, D. B. Estimating causal effects of treatments in randomized and nonrandomized studies. *J. Educ. Psychol.* **66,** 688–701 (1974).

5. Rosenbaum, P. R. & Rubin, D. B. The central role of the propensity score in observational studies for causal effects. *Biometrika* **70,** 41–55 (1983).

6. van der Laan, M. J., Polley, E. C. & Hubbard, A. E. Super learner. *Stat. Appl. Genet. Mol. Biol.* **6,** (2007).

7. Polley, E. & van der Laan, M. J. *SuperLearner in prediction*. (Division of Biostatistics, University of California-Berkeley, 2010).

8. Polley, E. C., Rose, S. & van der Laan, M. J. *Targeted learning: causal inference for observational and experimental data* (Springer New York, 2011).

9. Lee, B. K., Lessler, J. & Stuart, E. A. Improving propensity score weighting using machine learning. *Stat. Med.* **29,** 337–346 (2010).

10. Pirracchio, R., Petersen, M. L. & van der Laan, M. Improving propensity score estimators’ robustness to model misspecification using super learner. *Am. J. Epidemiol.* **181,** 108–119 (2015).

11. Ho, D. E., Imai, K., King, G. & Stuart, E. A. Matching as nonparametric preprocessing for reducing model dependence in parametric causal inference. *Polit. Anal.* **15,** 199–236 (2007).

12. Rubin, D. B. The use of matching and regression adjustment to remove bias in observational studies. *Biometrics* **29,** 185–203 (1973).

13. Rubin, D. B. Matching to remove bias in observational studies. *Biometrics* **29,** 159–183 (1973).

14. Cochran, W. G. & Rubin, D. B. Controlling bias in observational studies: a review. *Sankhyā Indian J. Stat. Ser. 1961-2002* **35,** 417–446 (1973).

15. Rosenbaum, P. R. & Rubin, D. B. Constructing a control group using multivariate matched sampling methods that incorporate the propensity score. *Am. Stat.* **39,** 33 (1985).

16. Rosenbaum, P. R. Optimal matching for observational studies. *J. Am. Stat. Assoc.* **84,** 1024–1032 (1989).

17. Rosenbaum, P. R. A Characterization of Optimal Designs for Observational studies. *J. R. Stat. Soc. Ser. B Methodol.* **53,** 597–610 (1991).

18. Bertsekas, D. P. *Linear network optimization: algorithms and codes*. (MIT Press, 1991).

19. Hansen, B. B. Full matching in an observational study of coaching for the SAT. *J. Am. Stat. Assoc.* **99,** 609–618 (2004).

20. Hansen, B. B. & Klopfer, S. O. Optimal full matching and related designs via network flows. *J. Comput. Graph. Stat.* **15,** 609–627 (2006).

21. Cochran, W. G. The effectiveness of adjustment by subclassification in removing bias in observational studies. *Biometrics* **24,** 295–313 (1968).

22. Rosenbaum, P. R. & Rubin, D. B. Reducing bias in observational studies using subclassification on the propensity score. *J. Am. Stat. Assoc.* **79,** 516 (1984).

23. Imai, K. & van Dyk, D. A. Causal inference with general treatment regimes. *J. Am. Stat. Assoc.* **99,** 854–866 (2004).

24. Rosenbaum, P. R. *Observational Studies*. (Springer-Verlag, 2002).

25. Hernán, M. A. & Robins, J. M. Estimating causal effects from epidemiological data. *J. Epidemiol. Community Health* **60,** 578–586 (2006).

26. Sekhon, J. S. *Multivariate and Propensity Score Matching Software with Automated Balance Optimization: The Matching Package for R*. (Social Science Research Network, 2008). Available at http://papers.ssrn.com/abstract=1009044. (Accessed 15th December 2015).

27. Diamond, A. & Sekhon, J. S. Genetic matching for estimating causal effects: a general multivariate matching method for achieving balance in observational studies. *Rev. Econ. Stat.* **95,** 932–945 (2012).

28. van der Laan, M.J. & Rubin, D. Targeted maximum likelihood learning. *Int. J. Biostat.* **2,** 11 (2006).

29. van der Laan, M. J. & Rose, S. *Targeted learning: causal inference for observational and experimental data*. (Springer New York, 2011).

30. van der Laan, M. J., Petersen, M. & Zheng, W. Estimating the effect of a community-based intervention with two communities. *J. Causal Inference* **1,** 83–106 (2013).

31. Caliendo, M. & Kopeinig, S. Some practical guidance for the implementation of propensity score matching. *J. Econ. Surv.* **22,** 31–72 (2008).

32. Stuart, E. A. Matching methods for causal inference: a review and a look forward. *Stat. Sci.* **25,** 1–21 (2010).

33. Harder, V. S., Stuart, E. A. & Anthony, J. C. Propensity score techniques and the assessment of measured covariate balance to test causal associations in psychological research. *Psychol. Methods* **15,** 234–249 (2010).

34. Stuart, E. A., Lee, B. K. & Leacy, F. P. Prognostic score-based balance measures can be a useful diagnostic for propensity score methods in comparative effectiveness research. *J. Clin. Epidemiol.* **66,** S84–S90.e1 (2013).

35. Petersen, M. L., Porter, K. E., Gruber, S., Wang, Y. & van der Laan, M. J. Diagnosing and responding to violations in the positivity assumption. *Stat. Methods Med. Res.* **21,** 31–54 (2012).

36. Freedman, D. *Statistical models: theory and practice* (Cambridge University Press, 2009).

37. Bouckaert, R. R. & Frank, E. *Advances in knowledge discovery and data mining* (eds. Dai, H., Srikant, R. & Zhang, C.) 3–12 (Springer Berlin Heidelberg, 2004).

38. Dietterich, T. Machine learning research: four current directions. *AI Mag.* **18,** 97–136 (1997).

39. R Core Team. *R: a language and environment for statistical computing*. (R Foundation for Statistical Computing, 2015).

40. Ho, D., Imai, K., King, G. & Stuart, E. MatchIt: nonparametric preprocessing for parametric causal inference. *J. Stat. Softw.* **42,** (2011).

41. Lendle, S. *tmlecte* (2015). Available at https://github.com/lendle/tmlecte. (Accessed 15th December 2015)

1. Simulations employing covariates with non-normal distributions produced very similar results and hence, are not reported. [↑](#footnote-ref-2)
